# Factors affecting adherence to disease-modifying therapies in multiple sclerosis: systematic review

**DOI:** 10.1007/s00415-021-10850-w

**Published:** 2021-10-21

**Authors:** Francesca Washington, Dawn Langdon

**Affiliations:** Department of Psychology, Royal Holloway, University of London, Egham, UK

**Keywords:** Adherence, Disease-modifying drugs, Multiple sclerosis

## Abstract

People with multiple sclerosis (MS) face challenges adhering to disease-modifying drug (DMD) treatment. Poor adherence to treatment reduces its clinical effectiveness which can adversely impact disease progression, MS-related hospitalisation, and mortality rates. Understanding the barriers to adherence is essential to addressing these issues in clinical practice and a consolidation of the literature had not yet been carried out. A systematic search was carried out using the electronic databases PsycINFO, and PubMed (Medline) using the search terms treatment compliance or treatment adherence and multiple sclerosis or MS. Studies included adults, with a diagnosis of relapsing–remitting MS (RRMS) (sample > 80% RRMS), taking a DMD. The studies used an adequate measurement of treatment adherence and analysed possible factors associated with adherence. A total of 349 studies were retrieved, of which 24 were considered eligible for inclusion. Overall adherence rates of the included studies ranged from 52 to 92.8%. Narrative synthesis revealed the most prevalent factors associated with adherence were age, gender, depression, cognition, treatment satisfaction, injection-site reactions, and injection anxiety. There was contradictory evidence for disability in association with treatment adherence. The findings should be used to inform the development of targeted patient support programs which improve treatment compliance. The review also highlights the opportunities for advancing research into treatment adherence in MS.

**Supplementary Information:**

The online version contains supplementary material available at 10.1007/s00415-021-10850-w.

## Introduction

Adherence to long-term treatment can be challenging for those suffering from a chronic illness, such as multiple sclerosis. In a widely cited report, the World Health Organisation (WHO) stated that only 50% of patients adhere to treatment recommendations [1]. It is thought that if patients are better able to adhere to treatment regimes in chronic disease, then this would have a greater impact on health outcomes than other therapeutic advances. Multiple sclerosis (MS) is a chronic auto-immune disease of the central nervous system which affects over 2.8 million people worldwide [2]. MS is characterised by demyelination, inflammation and neurodegeneration of the brain and spinal cord which can lead to significant cognitive and physical disability. There are different sub-types of MS based on the course of the disease and this effects prognosis and treatment options. Relapsing–remitting MS (RRMS) is the most common form of the condition and represents approximately 80–85% of initial diagnoses [3]. RRMS is marked by episodes of illness and disability known as relapses which are then followed by a period of remission. The clinical trajectory of MS is highly variable and shared-decision making about treatment is integral to effective patient care. Multiple sclerosis remains an incurable disease and pharmacological treatment aims to minimize the debilitating symptoms, slow progression, and protect the quality of life.

There have been remarkable advancements in the last 20 years in developing MS drug treatments known as disease-modifying drugs (DMDs) which slow the progression of the disease and reduce the rate of relapse [4]. Currently, there are at least 15 DMDs which have been approved for the treatment of MS in the UK [5]. “First-line treatments”, including the interferon injectables and glatiramer acetate, are offered to patients at the very first stage after RRMS diagnosis. In the last decade a number of oral and monoclonal antibody therapies for RRMS (“second line treatments”) have been developed, which are known to provide better efficacy than first-line treatments, but are associated with more adverse side effects [6]. Consequently, patients face a complex and multi-faceted decision when deciding the most appropriate disease-modifying therapy. A review of 24 studies which examined treatment adherence in MS found that adherence of disease-modifying drug treatment can be poor, and ranged between 41 and 88% [7]. Although effectiveness and compliance vary among the different DMD options, and personal preferences may be key, adherence is pervasively poor [6]. Despite the development of oral DMD’s, which were hoped to mitigate adherence issues, one in five patients with MS do not adhere to, and one in four discontinue, daily oral DMDs before 1 year [8]. If adherence to treatment is poor, medication will have limited clinical effectiveness and risks increased disease activity [9]. Furthermore, the cost of specialist multi-disciplinary care increases with disease progression and those who do not effectively adhere to treatment are more likely to require hospitalisation due to MS complications [10].

Medication compliance or adherence is defined as “the extent to which a patient acts in accordance with the prescribed interval and dose of a dosing regimen” [11]. This is often operationalised in research as the percentage of doses taken in relation to what was prescribed over a set time period. This is a highly specific aspect of the inclusion criteria of this review so only studies which adequately measured adherence rates were included. The measure of persistence to drug therapy can be defined as conforming to the recommended treatment over a set period of time. A recent systematic review analysed factors associated with treatment discontinuation of second wave DMDs and is, therefore, an analysis of persistence rates rather than adherence [12]. Factors related to adherence may differ to those of persistence, therefore a synthesis of studies focusing solely on adherence is warranted [13]. Measuring adherence provides a more comprehensive analysis of a patient’s ability to consistently follow an often arduous and time-consuming medication regime [7]. Poor adherence has been associated with patient factors, disease management and treatment regimes [14].

Adherence to drug treatment in MS cannot be quantified using biological markers, therefore measuring adherence is often largely reliant on patients’ self-report [15]. Patients are asked to keep a record of how many doses they have taken over a fixed period of time and this is used to calculate the missed dose ratio (MDR), which is the proportion of missed doses out of the number of prescribed doses. Adherence can also be measured through pharmacy claims-based calculations, such as the medication possession ratio (MPR) or the proportion of days covered (PDC). This refers to the number of days the patient has access to their prescribed medication and is considered a proxy for adherence [11]. More objective measures of adherence have recently been developed, which involve an electronic device tracking self-administered injections [16]. The device is connected to an electronic database which captures and records when the patient performed the injection. Adherence studies often use categorical variables to define adherence, typically using a cut off of > 80% to assign patients to adherent or non-adherent groups and this can be used to determine the overall rate of adherence for the study population.

Adherence to treatment in multiple sclerosis is crucial to optimising patient care and managing the long-term prognosis of people with MS. Developing a strong evidence base for the key factors associated with adherence will help inform the development of targeted interventions such as patient support programs which are focused on treatment compliance. The effectiveness of these interventions could be subsequently evaluated by measuring adherence rates and health-related outcomes specific to this population. The aim of the current review was to provide a synthesis of the factors associated with adherence to disease-modifying drugs in the treatment of multiple sclerosis.

## Method

### Search strategy

A comprehensive literature search was conducted according to the ‘PRISMA’ (preferred reporting items for systematic reviews and meta-analysis) statement [17]. Studies were identified using the electronic databases PsycINFO, and PubMed (MEDLINE) and searches were completed on 20th June 2020. Searches were conducted using the following terms as keywords in titles and abstracts: “treatment compliance” OR “treatment adherence” AND “multiple sclerosis” OR “MS”.

The studies included in existing systematic reviews related to drug adherence in multiple sclerosis were screened for eligibility for this review [7, 17]. Reference lists of the studies included in this review were also screened for eligible studies. In addition, internet searches were carried out to identify any full-text publications which had not been identified by the database search.

### Study eligibility

Studies were limited to those written in English and published in peer-reviewed journals. Studies were included if they assessed factor/s related to the adherence of disease-modifying drug treatments for multiple sclerosis and reported quantitative data. Studies were only included if participants had a confirmed diagnosis of relapse-remitting multiple sclerosis, or included a sub-group of whom > 80% had the relapse-remitting form, a criteria used in a recent review [18]. Studies which included both clinically isolated syndrome and MS patients, but failed to specify the proportion of each, were excluded. The studies included participants who were 18 years of age and older and were prescribed an FDA-approved disease-modifying drug. Studies identified used a non-randomized, observational design which was either cross-sectional or longitudinal. Studies were excluded if they did not employ an adequate measure of adherence, or they focused on persistence measures (discontinuation or switching rates) in isolation. The review did not include studies which solely assessed the impact of a patient intervention on adherences rates or those which exclusively looked at factors contributing to reinitiation of drug therapy following a period of non-compliance. The selection of all included studies was examined by a second reviewer, and this resulted in 3 studies being excluded.

### Data extraction

One reviewer (FW) extracted data from the studies directly into a table made specifically for the current review and this was examined and verified by a second reviewer (DL). Study characteristics which were extracted included: participant eligibility criteria, sample size and prescribed DMD, adherence measurement, adherence rate and the key findings. It was not possible to carry out a meta-analysis of the included studies due to methodological diversity, therefore a narrative synthesis of the key findings was conducted.

### Quality assessment

The quality of each study and risk of bias was assessed using an adapted AXIS Critical Appraisal tool which included 12 items [19]. For each item a (*) star was awarded if the criteria had been adequately met and a (–) minus sign if it had not been adequately met.

## Results

### Study selection

**Fig. 1:**
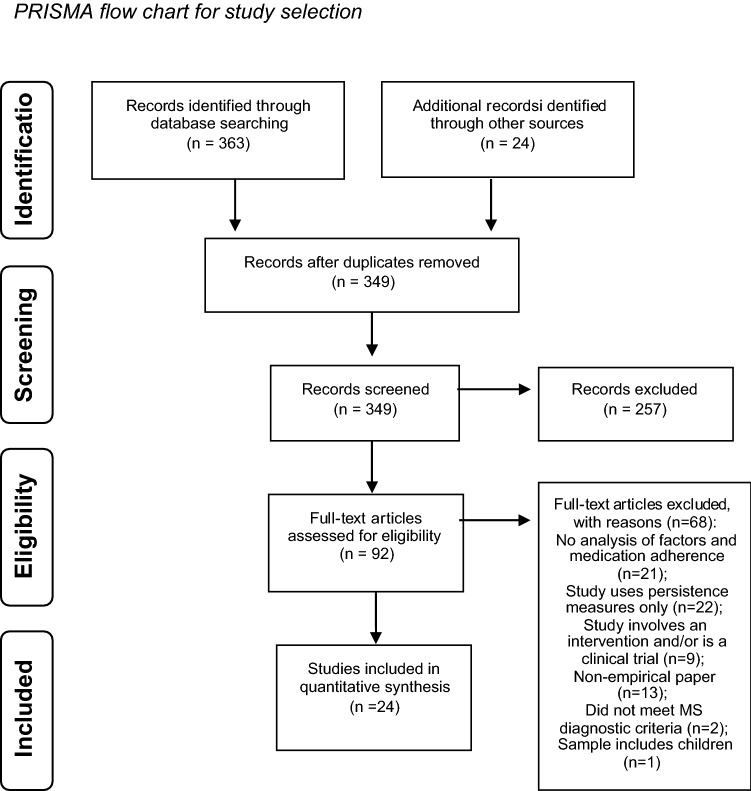
PRISMA flow chart for study selection

A total of 24 studies were included in the current review. Eight studies did not specify the participants’ MS subtype. These papers recorded the disease-modifying drugs that the participants were prescribed, which were only licensed for patients with relapsing–remitting MS [20]. Therefore, it can reasonably be assumed that these studies included participants with relapsing–remitting MS. The selection process is illustrated in Fig. 1.

### Data extraction

The relevant data was extracted from the 24 included studies and can be found in the Supplementary Information, Table 1.

### Quality assessment

Of the 24 studies, four adequately met all the 12 evaluated items and no studies were given a minus sign for more than three of the items (Table 1). Most of the studies received one or two minus signs (*n* = 14). All included studies used the appropriate design for their research aims. No studies were excluded from the review following the quality assessment.

**Table 1:** Results of quality assessment

	Study design	Justified sample size	Representative sample	Appropriate variables for aim	Valid measures	Misclassification bias minimised	Basic data reported	Adequate non-response rate	Justified conclusions	Limitations reported	No conflicts of interest (funding)	Ethical approval	Number of minus signs
Arroyo et al. [32]	*	–	*	*	*	*	*	–	*	*	–	*	3
Bruce et al*.* [24]	*	–	–	*	*	*	*	*	*	*	*	*	2
Devonshire et al*.* [31]	*	*	–	*	*	*	*	Nr	*	*	–	*	2
Erbay et al*.* [29]	*	–	–	*	*	*	*	Nr	*	–	*	*	3
Evans et al*.* [9]	*	*	*	*	*	*	*	*	*	*	*	*	0
Hao et al. [15]	*	*	–	*	*	*	*	*	*	*	*	*	1
Higuera et al*.* [22]	*	*	*	*	*	*	*	*	*	*	*	*	0
Jongen et al*.* [36]	*	–	–	*	*	*	*	Nr	*	*	*	*	2
Koltuniuk and Rosinczuk [35]	*	–	–	*	*	*	*	Nr	*	*	*	*	2
Koskderelioglu et al*.* [30]	*	–	–	*	*	*	*	Nr	*	–	*	*	3
Lahdenperä et al. [25]	*	*	*	*	*	*	*	*	*	*	–	Nr	1
Li et al*.* [5]	*	*	*	*	*	*	*	*	*	*	–	*	1
Lugaresi et al*.* [37]	*	*	–	*	*	*	*	*	*	*	–	*	2
Mckay et al*.* [26]	*	*	*	*	*	*	*	–	*	–	*	*	2
Munsell et al*.* [23]	*	*	*	*	*	*	*	*	*	*	*	*	0
Ožura et al*.* [38]	*	–	*	*	*	*	*	*	*	*	*	*	1
Paolicelli et al. [16]	*	–	–	*	*	*	*	*	*	*	*	*	2
de Seze et al. [33]	*	–	*	*	*	*	*	–	*	*	*	*	2
Siegel et al*.* [41]	*	–	–	*	*	*	*	–	*	*	*	*	3
Thach et al*.* [27]	*	*	–	*	*	*	*	*	*	*	*	*	1
Treadaway et al*.* [34]	*	*	*	*	*	*	*	*	*	*	*	*	0
Tremlett et al. [21]	*	–	–	*	*	*	*	–	*	*	*	*	3
Turner et al. [42]	*	–	–	*	*	*	*	*	*	*	*	*	2
Zecca et al*.* [28]	*	–	–	*	*	*	*	Nr	*	*	–	*	3

(*) adequately met criteria, (−) = did not adequately meet criteria, *Nr* not reported

### Study characteristics

There were nine studies carried out in the USA, two in Turkey, two in Canada, two in Italy and one in each of Australia, Finland, France, Netherlands, Poland, Slovenia, Spain, and Switzerland. One study recruited participants from 22 countries around the world. Of the 24 studies, 13 received some funding from a pharmaceutical or biotechnology company. There were three studies funded by the National MS society, two were funded by Veterans Research, two were funded by health research bodies, and one study was funded by a government organisation. Three studies that had no external funding.

The sample size of the studies varied from 53 to 17,599 participants and the majority of participants were recruited through out-patient clinics or neurology treatment centres (*n* = 17). There were seven studies which recruited participants through an MS registry or health databases. One study also recruited participants through the US National MS Society and the media [21].

### Disease-modifying drug treatment

There were 23 studies which examined adherence to those who were prescribed first-line treatments which were either an oral or self-injectable drug. Of these studies, three studies also included second-wave treatments such as ‘fingolimod’ [5, 22, 23]. There was only one study which stated they examined adherence to ‘natalizumab’ which is a medication that requires intravenous infusion under medical supervision in a hospital [22]. One study did not provide details of the disease-modifying therapy their participants were prescribed but as adherence was measured through pharmacy claims this will not include a drug treatment given in hospital.

### Adherence measurement

There was considerable heterogeneity in the methods used to calculate treatment adherence and quantitative cuts-off used to define adequate adherence. There are also some studies which used more than one adherence measure. There were 10 studies which used a longitudinal design and measured adherence across different time points, and the remaining 14 measured adherence across a single time-point. Of the 24 studies, 15 of them captured participants’ adherence using self-report. These studies measured adherence through either missed doses or taken doses over a fixed time period ranging from 2 weeks to 6 months. There were four studies that assessed adherence through electronic monitoring. Bruce et al*.* [24] also used an electronic monitoring system which recorded needle disposal, alongside the self-reported MDR. Six studies used pharmacy-based claims to estimate adherence: Evans et al*.* [9], Lahdenperä et al. [25] and Li et al*.* [5] used the proportion of days covered (PDC) and Higuera et al. [22], Mckay et al. [26] and Munsell et al. [23] used medication possession ratio (MPR). These studies measured adherence across different fixed time intervals.

### Design and analytic strategy

All of the studies assigned participants to categorical groups related to adherence, these were most commonly referred to as “adherent” or “non-adherent”, or similar. However, there were some differences in the cut-offs used. Only one study used continuous data for additional analysis to examine the effect of different factors related to adherence (Bruce et al. [24]). Of the 15 studies which used self-report to measure adherence, seven categorised participants as adherent if they missed at least one dose in the set time period. Different DMDs are taken at different intervals; five studies accounted for this, and standard weightings were used to calculate the missed dose ratio.

Most of the included studies used questionnaires or scales to quantify potential predictors of adherence, alongside capturing participants’ sociodemographic information and the clinical characteristics of their MS. Several studies also gave participants surveys and provided qualitative comments or answered multiple-choice questions. This information was then used to carry out descriptive analysis of their perception of the contributing factors to non-adherence.

### Adherence rates

The adherence rates of the studies range from 52 to 92.8%. The overall mean rates of adherence were pooled together based on the adherence measurement that was used by the study. It is important to note that the studies measured adherence across different time periods and used varying cut-offs. In addition, it was not possible to provide weighted means of adherence to the different disease-modifying drugs. Baseline adherence rates were used in the studies which had a longitudinal design. For the six studies which used pharmacy-based claims to measure adherence, either through the MPR or POC calculation, the mean rate of adherence was approximately 76.9%. The four studies which used an objective adherence measurement had a mean adherence rate of 80.55%. Finally, the mean rate of adherence of the self-reported studies was 74.0%.

### Factors associated with adherence

Several factors were significantly associated with adherence rates or were identified through descriptive analysis. The factors were systematically coded and used to generate descriptive themes.

#### Gender

All of the included studies analysed how sociodemographic characteristics related to adherence rates, and gender and age were the most consistently related. The review found four studies which showed that men had better treatment adherence than women and given that MS is more prevalent amongst females, this is of particular importance. Higuera et al. [22] used multi-variate probit models to identify factors associated with treatment adherence and found a correlation between gender and adherence and this showed that women had an estimated probability of adherence that was 5.5 percentage-points lower than men. Similarly, Li et al. [5] conducted a multi-variable regression which revealed that men had higher odds of being adherent than women (OR: 1.15; 95% CI 1.11–1.19) and Munsell et al. [23] also found that the male gender was more likely to be associated with adherence (OR 1.3; 95% CI 1.085–1.335, *p* = 0.0005). Lahdenperä et al. [25] also found male gender was significantly associated with adherence (OR = 1.160, 95% 1.034–1.300, *p* = 0.0112).

#### Age

Six studies found older age to be positively associated with adherence, suggesting age is a strong predictor. Paolicelli et al. [16] reported that participants aged 26–40 were statistically more adherent than both participants aged $\le$ 25 and those aged > 40 (*p* = 0.006) but older age was found to be a more consistent predictor of adherence. Thach et al. [27] found adherence improved with older age (*p* = 0.011). Zecca et al. [28] also found that older age was associated with better adherence to medication (*p* = 0.008) and Higuera et al. [22], using the multi-variate probit model, found that those aged 45 and older were more likely to be adherent compared to patients aged between 18 and 34. Similarly, Munsell et al. [23] found that age groups older than 18–34 were more adherent to treatment (ORs 1.220–1.331; *p* < 0.01). Older age was also associated with adherence in Lahdenperä et al. [25] study (*p* < 0.0001). There is a lack of theoretical understanding in the literature about why age may predict adherence, particularly as confounding variables such as symptom stability and disability are often controlled for in studies.

#### Education

Erbay et al. [29] found that married patients with children showed statistically lower treatment adherence levels (*p* < 0.05). Koskderelioglu et al. [30] found that 55% of the more highly educated group was non-adherent, compared to only 3.6% in their less-educated group, a statistically significant difference (*p* = 0.007). Consistent with this, Devonshire et al. [31] found that participants with a higher education were less likely to be adherent than those who did not go to college or finish their degree (*p* = 0.02).

#### Disease profile

Devonshire et al. [31] found that adherent participants had been diagnosed with multiple sclerosis for a significantly shorter period of time (median = 6.0 years) than non-adherent participants (median = 7.0 years; *p* < 0.001). Furthermore, adherent participants had also been taking their current disease-modifying drug for a shorter period of time (median = 30.0 months) than non-adherent participants (36.0 months; *p* = 0.005). Similarly, McKay et al. [26] found that disease duration ($\ge$ 5 years) was significantly associated with non-adherence, after adjusting for potential confounders (OR = 2.23; 95% CI 1.10–4.52).

Arroyo et al. [32] used a questionnaire completed by each participant’s treating neurologist to assess factors related to adherence and found that a number of MS relapses was associated with adherence (66.8%) which was the most common reason cited at follow up (2 years).

#### Disease information and management

In the de Seze et al. [33] study adherence was found to be significantly higher in those patients who were well informed about their condition and treatment (*p* = 0.035). Erbay et al. [29] reported that adherent patients were significantly more satisfied with their treatment than non-adherent patients (*p* = 0.0003) and Treadaway et al. [34] also found that non-adherent patients reported less satisfaction with their treatment compared with those who were adherent (*p* < 0.001). Koltuniuk and Rosinczuk [35] reported a similar outcome; one of the most frequently mentioned reasons participants gave for not taking their medication was dissatisfaction with treatment, although this was not found to be statistically significant between the adherent and non-adherent groups. Hao et al. [15] used the TSQM Global Satisfaction questionnaire which showed that there were higher scores on this measure in the high adherence group compared to the low adherence group. Finally, Jongen et al. [36] examined the impact of different aspects of care on adherence and found that those patients who received more care at home and more informal care demonstrated better adherence (*p* = 0.007 and *p* = 0.020, respectively). Similarly, the duration of these care activities were also positively associated with adherence (*p* < 0.05).

#### Disability

Six studies assessed participants’ disability using the Expanded Disability Status Scale (EDSS) with mixed findings. Four studies found that higher disability was associated with non-adherence, however, two studies found that those with lower disability were more likely to be non-adherent. Koskderelioglu et al. [30] found that a higher EDSS score (i.e., greater disability) had a negative effect on treatment adherence (*p* < 0.0001). Paolicelli et a. [16] demonstrated a similar finding, and a logistic regression analysis revealed that an EDSS score of $\ge$ 4 reduced the probability of being adherent, after adjusting for age group (OR = 0.29; CI 0.1–0.8; *p* = 0.015). In addition, Li et al. [5] found that ‘disability’ as the current or original Medicare entitlement reason was associated with lower odds of being adherent across all age groups. Similarly, Hao et al. [15] found that those with intermediate or high adherence rates had significantly better scores on the EDSS and MSIS physical scales (less comparable disability) compared to their low adherence group (*p* < 0.05). However, Zecca et al. [28] found that after performing an ordinal regression analysis, that a greater EDSS score (greater disability) was significantly associated with higher objective adherence (OR = 1.937; CI 1.197–1.937; *p* = 0.008). Mckay et al. [26] found a similar finding and reported that a lower EDSS score was significantly associated with non-adherence, and those with mild disability (score of 0–2.5) were more likely to be non-adherent than those with moderate disability (score of 3.0–5.5) (OR: 1.80; 95% CI 1.06–3.04).

Psychological and behavioural factors.

#### Cognition

A total of nine studies found that self-reported memory problems or forgetfulness were associated with poorer adherence or missing doses. Descriptive analysis carried out by Arroyo et al. [32] revealed that forgetfulness was the most common reason participants offered at baseline for not administering their injections (70.3%). It was also the second most cited reason for missed injections at year 1 (42.1%) and year 2 (32%). In the Devonshire et al. [31] study ‘forgetfulness’ was also cited as the most common reason for non-adherence (50.2%). de Seze et al. [33] explored reasons for skipping and stopping injections and they found that forgetfulness accounted for the greatest percentage (38.7% of the 93 participants). Participants in the study by Treadaway et al. [34] listed forgetting as the most common reason for missing their injection (58%). Forgetfulness was the second most cited reason for non-adherence in the Lugaresi et al. study [37] (20.7%). ‘Memory problems’ were the most common self-reported reason for missing an injection in the descriptive study carried out by Erbay et al. [29]. Koltuniuk and Rosinczuk [35] used a disease-modifying therapy barriers questionnaire to quantify the reasons for non-adherence, which asked participants to rate their reason on a four-point scale: 43.39% said memory problems were *not important at all*, 16.98% said it was *a little important*, 20.75% found it was *moderately important* and 18.86% found it was *extremely important*. In total, more than half the sample (56.59%) stated memory problems as important. Mckay et al. [26] performed a multivariable analysis, adjusting for potential confounders, and this revealed that perceived moderate or severe cognitive difficulties were significantly associated with non-adherence (OR: 2.14; 95% CI 1.23–3.75). Self-reported memory problems were also one of the most common reasons for non-adherence in the Ožura et al. [38] study.

Two studies used quantitative scales to measure cognition; Bruce et al. [24] found that retrospective self-reported adherence was associated with both worse prospective memory (*r* = − 0.28, *p* < 0.05) and poorer delayed list recall (*r* = − 0.29, *p* < 0.05). Between-group analyses of adherence revealed that poor adherers demonstrated poorer performance on a test of prospective memory compared to adequate adherers. Poor adherers also recalled fewer words after a delay (mean = 8.29 $\pm$ 3.06; *t*(53) = 2.09, *p* < 0.05). Devonshire et al. [31] used the Multiple Sclerosis Neuropsychological Questionnaire (MSNQ) which is a reliable self-reported screening tool for cognitive impairment. The study found that adherent patients had a significantly lower median MSNQ score than non-adherent patients (*p* < 0.0001). Inflammatory demyelination can result in cognitive deficit in up to 75% of people with MS and can be present in the very early stages of the condition [39]. Therefore, unsurprisingly, these difficulties often manifest as memory issues/forgetfulness which make it difficult for patients to consistently adhere to a treatment regime.

#### Depression/quality of life

A diagnosis of depression, symptoms of depression or at least one psychiatric disorder were associated with poorer adherence across five studies. Bruce et al. [24] found that patients with at least one psychiatric disorder showed significantly worse adherence, evident on all four adherence measurements used by the study (three self-report measures (*p* < 0.001) and one objective measure (*p* = 0.001)). Worse scores for retrospective self-reported adherence were also associated with increased anxiety symptoms (*p* < 0.01). There were four studies which found that depression or depressive symptoms had an association with adherence rates. Koskderelioglu et al. [30] found that higher scores on the Beck Depression Inventory (scale used to evaluate symptoms of depression) were associated with non-adherence (*p* = 0.006). The same scale was used by Treadaway et al. [34], who also demonstrated higher scores for depression in the non-adherent group (*p* = 0.0009). Similarly, Higuera et al. [22] also found that those patients who had been diagnosed with depression in the previous year were 5.5 percentage points less likely to be adherent in the current year of the study, and this was found to be marginally significant. Munsell et al. [23] also found that depression was associated with a lower likelihood of adherence (OR 0.618; 95% CI 0.511–0.747; *p* < 0.0001). Neuropsychiatric comorbidities are prevalent amongst people with MS, a recent meta-analysis demonstrated consistent evidence for high prevalence rates of depression (31%) and anxiety (22%) in MS patients [40].

Devonshire et al. [31] analysed the quality of life against adherence rates and found that adherent patients had a better quality of life scores. Analysis of specific domains found that adherent patients had significantly higher scores on relationship with family (*p* < 0.0001), sentimental and sexual scales (p = 0.0068) and activities of daily living (*p* = 0.0021). Treadaway et al. [34] also looked at the quality of life domains and found differences between the emotional well-being scores in the adherent and non-adherent groups. The domains of statistical significance were: emotional problems (*p* < 0.0001), emotional well-being (*p* = 0.0012), social function (*p* = 0.0227), overall quality of life perception (*p* = 0.0001) and mental health composite scores (*p* < 0.0001). Similarly, Hao et al. [15] reported that patients in the high adherence group had statistically significantly better means on the MS impact scale psychological scores than those in the low adherence group (*p* = 0.05). In relation to psychological support, Siegel et al. [41] conducted a multivariate logistic regression, controlling for type of DMD, length of time on DMD and MS-associated disability, and found that supportive qualities of the caregiver relationship significantly predicted a better adherence (OR = 3.58, 95% CI = 1.09–11.80).

#### Alcohol consumption

Tremlett et al. [21] found that increased alcohol consumption (frequency and amount) was associated with an increased risk of missing a few or multiple doses. In the adjusted model, those who drank on average 1–2 or 3 + standard drinks per session were 5–7 times more likely to miss a few doses (*p* = 0.008) and up to 14 times more likely to miss multiple doses (*p* = 0.008), than those who did not drink. Similarly, Mckay et al. [26] multivariate analysis revealed that those who were alcohol-dependent were twice as likely to be non-adherent than those who were not (OR = 2.14, 95% CI 1.23–3.75).

#### Medication-specific issues

Several studies captured medication-specific reasons for participant’s non-adherence to treatment, and injection anxiety (*n* = 3) and injection-related reactions (*n* = 4) were commonly reported. Arroyo et al. [32] carried out a descriptive analysis of reasons participants gave for lack of adherence and found that the second most common reason was injection-related reactions (43.2%) at baseline (which included tired of self-injection, skin reactions, needle phobia, injection site pain, not feeling the need to inject and nobody available to administer the injections). Injection-related reactions were also the most common reason for lack of adherence at year 1 and year 2 (89.5% and 72%, respectively) in this study. Devonshire et al. [31] also found that injection-site reactions were the second most common reason for non-adherence and was reported by 32% of participants. The reactions included injection anxiety, skin reaction, not feeling the need for every injection and having nobody available to administer the injection. Similarly, Paolicelli et al. [16] found that injection-site reactions were the third most common reason for missing doses (20.9%). Higuera et al. [22] reported that those taking self-injectable medications, whose most common side effect was injection site reactions, were 9.1 percentage- points less adherent than those whose common side effect was flu-like symptoms. Injection anxiety was explored by Turner et al. [42], who found that those who were nonadherent at any follow-up time point endorsed significantly higher injection anxiety at baseline, after adjusting for demographic characteristics, MS disability, medication type and time on DMD (*t*(88) = 2.65, *p* < 0.01).

Three studies found that participants reported systemic physiological effects of taking their medication and attributed this to their non-compliance. Devonshire et al. [31] found that experiencing flu-like symptoms was the most common reason for non-adherence by patients on IM IFN $\upbeta$-1a (28.9%), although this was cited less often by patients taking other disease-modifying drugs. Similarly, Paolicelli et al. [16] found that flu-like syndrome was the most cited reason for increased missed doses (55.8%), hematological side effects were also cited (7.2%). In addition, Arroyo et al. [32] showed that the participants’ neurologists highlighted factors related to adverse effects of treatment in relation to lack of adherence (88.9% at baseline and 92.9% after 2 years).

One study highlighted issues related to the practicalities of taking medication at home. Koltuniuk and Rosinczuk [35] reported that participants in the non-adherent group cited missing injections due to being away from home, and that taking the drug interfered with daily activities.

## Discussion

### Main findings

The review provides a comprehensive synthesis of the factors associated with treatment adherence in MS. Due to the considerable methodological heterogeneity across the studies, a narrative synthesis of the findings was performed. The review process also highlighted some of the main challenges associated with measuring adherence in this population. No studies were removed following quality assessment and the overall quality of the studies was deemed acceptable particularly in the context of the challenges related to measuring treatment adherence in MS. The adherence rates of included studies ranged from 52 to 92.8%, which are comparable to a recent review of adherence rates [7]. This substantiates the evidence that adherence to disease-modifying drugs in the treatment of MS continues to be an unmet need in the management of this condition.

The review found that gender, age, depression, cognition, treatment satisfaction, and treatment side effects were the most prevalent factors associated with adherence to treatment. The potential risk factors for poor adherence identified in this review have implications for clinical practice, both directly in neurology outpatient clinics and more broadly when designing patient support programs. When patients are initially deciding about treatment, it is important that neurologists are aware of the potential barriers to adherence. Clinicians may want to conduct a risk assessment of the potential variables which may influence adherence to consider targeted interventions [43]. Treatment satisfaction may be maintained through an open and trusting relationship between the patient and their healthcare team, with clear communication of the risks and benefits of prescribed DMDs, and appropriate management of treatment expectations. The emotional well-being of patients with MS should be continually monitored and specific mental health interventions may be warranted to improve compliance. Addressing cognitive difficulties which affect adherence is likely to be challenging, particularly when a patient’s cognition worsens over time, but efforts to empower patients to use their own tools (e.g., memory aids) to foster adherence should be used in the first instance. For fixed patient characteristics such as age and gender, healthcare services must adopt a patient-centred approach when addressing issues of compliance.

Patient support programs designed to improve compliance have been found to have a positive impact on adherence rates of DMDs [44]. They have also been shown to reduce the number of relapses and healthcare costs due to improved treatment adherence [45, 46]. However, adjusting human behaviour is challenging and improving adherence is a complex and dynamic process. As evidenced by the review, there are a range of different factors which may affect adherence. A Cochrane review has shown that a multifactorial approach is more effective in improving treatment adherence in chronic disease [47].

### Limitations

There are several limitations to this review which are inherent to the review process itself and across the included studies. The findings could not be statistically analysed, therefore, it is difficult to draw firm conclusions from the findings of this review. The review was also subject to positive bias reporting as only the statistically significant findings are presented and did not include a description of the null findings. There are also issues of bias within the included studies which effects the overall bias of the review. Several studies received some funding from a pharmaceutical or biotechnology companies and six of these were deemed to be a conflict of interest. Industry funding can present a source of bias as they may support a particular agenda and be influential at various stages of research design and implementation [48]. The research was also carried out in different countries which have varied healthcare systems, and this has implications for drug adherence.

Measuring compliance to treatment in MS is challenging in the absence of a psychometrically valid adherence measure. The review includes studies which used different tools to measure adherence such as self-reported data, pharmacy-based claims, and electronic monitoring, which have their own limitations. Studies which used a self-report measure are more likely to appeal to patients who show good treatment compliance, and they may also under-report their non-adherence due to social desirability. Several studies used pharmacy-based claims to measure adherence and this measure assumes compliance but does not guarantee it. There was also considerably discrepancy between the sample sizes of the studies; the smaller scale studies were from self-reported data and the larger ones used pharmacy-based claims. Therefore, this study contains a bias of results towards methodological flaws specific to pharmacy-based claims studies. The included studies examined adherence to several different disease-modifying drugs, and this increases the clinical heterogeneity of the review. These drugs carry different risk/benefit profiles and require different routes and frequencies of administration (including oral tablets, injectables and intravenous injection under medical supervision). Studies did not provide separate analyses for different disease-modifying drugs prescribed, although 6 studies did adjust for the different frequencies of administration. It is possible that significant drivers of adherence within sub-groups have been masked by the grouped data.

### Future directions

The study selection process highlighted the inconsistent operational definitions of adherence and persistence used in MS research. It is important for researchers to provide a clearer justification and understanding of these concepts and to use an adequate measure of adherence. The studies which adequately measured adherence which were included in the review have considerable methodological and clinical heterogeneity. The significant variability in adherence rates across studies may reflect this heterogeneity, therefore there is a need to develop a reliable, standardised measure of adherence. In the absence of this, studies should use more than one measure of adherence to increase ecological validity. In addition, the consistent use of the same outcome measures to quantify specific determining factors of adherence would improve the validity of findings. These methods of standardization would enable researchers to carry out a meta-analysis of the findings to draw more accurate and reliable conclusions. It would be useful to conduct longitudinal research across different time points to capture a better understanding of adherence across the course of the disease. Studies should also recruit participants who are prescribed second and third-line treatments so these can be separately systematically reviewed and then compared with first-line treatments. These findings would strengthen the evidence base for developing large-scale patient support programs and tailored interventions designed to improve compliance.

## Conclusion

Poor adherence to disease-modifying drug treatment in multiple sclerosis remains a challenge in clinical practice and has an adverse impact on prognosis. The current review has substantiated research into the factors associated with adherence, which includes gender, age, depression, cognition, treatment satisfaction and medication-specific issues. Priorities for future research include addressing the methodological and conceptual limitations of previous studies which will enable researchers to carry out meta-analyses. In an ageing population, the management of multiple sclerosis is likely to further contribute to a growing burden on healthcare services due to age-related comorbidities and further complications to disease management. Therefore, addressing issues related to adherence, and delivering interventions that improve compliance to treatment, is critical to minimising the personal and economic impact of the disease.

## Supplementary Information

Below is the link to the electronic supplementary material.

Supplementary file1 (DOCX 28 kb)
