# Supplementary material for: Factors affecting adherence to disease-modifying therapies in multiple sclerosis: systematic review
Source: J Neurol. 2021 Oct 21;269(4):1861–72. doi: 10.1007/s00415-021-10850-w (PMC8940867; doi:10.1007/s00415-021-10850-w)
Supplement: Supplementary file 1 — Supplementary file1 (DOCX 28 kb) [file 415_2021_10850_MOESM1_ESM.docx]

**Table 1**

Descriptive information of the included studies

| **Author (Date)** | **Participant eligibility criteria** | **Sample size and prescribed DMD** | **Adherence measurement** | **Adherence rate** | **Key findings** |
| --- | --- | --- | --- | --- | --- |
| **Arroyo et al. (2011)** | $\geq$18 years old  Diagnosis of RRMS only,  Taking 1 DMD for >6 months before enrolment | IM IFN$\beta$-1a (n=56);  IFN$\beta$-1a 22$\mu g$  (n=43);  IFN$\beta$-1a 44$\mu g$  (n=54);  IFN$\beta$-1b  (n=49);  GA  (n= 52) | Determined by self-report and neurologist report.  Adherent if did not miss a DMD injection taken in past 4 weeks  (collected at baseline, year 1 and year 2) | Baseline: 85.4%  Year 1: 86.6%  Year 2: 82.4% | - At baseline, most common reason for lack of adherence was forgetting to inject (73%), then injected-related reactions (43.2%) - At year 1 and 2 the most common reason for lack of adherence was injection-related factors (89.5 and 72%) followed by forgetting to dose (42.1% and 32%) |
| **Bruce et al. (2010)** | $\geq$18 years old  Diagnosis of RRMS only, self-injecting a DMD for at least 2 months | SC GA (n=45);  IM IFN$\beta$-1a (n=3);  SC IFN$\beta$-1b (n=7) | Three types of adherence measurement:  -Medication event monitoring system (calculated by percentage of days not covered by DMD).  -Self-reported adherence for 8 weeks retrospectively and prospectively (percentage of self-reported missed doses)  Adherent <10% missed doses. | 73% were defined as having good/adequate adherence (<10% missed doses) | - Poor adherence was associated with memory difficulties, anxiety, depression, neuroticism, and low conscientiousness |
| **Devonshire et al. (2011)** | $\geq$18 years old  Diagnosis of RRMS only.  Taking 1 DMD for at least 6 months | IM IFN$\beta$-1a (n=764);  IFN$\beta$-1a 22$\mu g$  (n=32);  IFN$\beta$-1a 44$\mu g$  (n=41);  IFN$\beta$-1b  (n=571);  GA  (n=36) | Determined by self-report.  Adherent if did not miss a single injection in the past 4 weeks | 75% | - Reasons for non-adherence was forgetting to administer the injection (50.2% and other injection-related reasons (32%) - Adherent patients reported better QoL and fewer neuropsychological issues, shorter duration of disease and shorter duration of therapy compared to non-adherent |
| **Erbay et al. (2018)** | $\geq$18 years old  Diagnosis of MS (93.4% RRMS, 6.6 SPMS).  Taking DMD for at least 1 month  EDSS score $\leq$6.5 | 198  IFN$\beta$-1a  (10.6%);  IFN$\beta$-1a  (25.8%);  IFN$\beta$-1b (28.3%)  GA (35.4%) | Determined by self-report  MDR calculated in a 1-month period.  Adherent if did not miss 1 injection | 59.6% | - Adherence was significantly associated with patients’ marital status - Adherence was significantly related to chronic disease and satisfaction with treatment - Non-adherence was related to memory problems, being away from home and side effects |
| **Evans et al. (2016)** | $\geq$18 years old  MS confirmed diagnosis (unspecified type)  In receipt of a prescription dispensation for a first-line DMD | 4830  SC IFN$\beta$-1a (28.5%)  IM IFN$\beta$-1a (14.2%)  IFN$\beta$-1b (31.4%)  GA (25.8%) | Estimated using the proportion of days covered (PDC)  PDC$\geq$80% was considered adherent | 76% | - Individuals with more than 4 physician visits during the year prior to first DMD being dispensed had more optimal adherence then those who attended fewer visits (0-3) - Age, sex and socioeconomic status were not associated with adherence |
| **Hao et al. (2017)** | $\geq$18 years old  MS confirmed diagnosis (unspecified type)  At least one medication indicated for MS | 681 | Prospective analysis was determined by self-report.  Adherence was classified as: low<80%, intermediate 80-99% and high 100% | 80% (80% adherence rate or greater) | - Patients with intermediate and high self-reported adherence had significantly better mean scores for disability, MSIS function and TSQM global satisfaction |
| **Higuera et al. (2016)** | $\geq$18 years old  MS confirmed diagnosis (unspecified type)  Had a prescription filled for an MS DMD in any year | 698  IM IFN$\beta$-1a  IFN$\beta$-1a  IFN$\beta$-1b  GA  Fingolimod  Teriflunomide  Dimethyl fumarate  Natalizumab | MPR calculated  MPR $\geq$80% considered adherent | 87.2% | - Women were less likely to be adherent and patients aged 45 years and older were more likely to be adherent - Those who took self-injectable medications whose most common side effect was injection site reactions were less adherent than those who took any other type of DMD, and other injectables   Depression had a significant impact on lower adherence rates |
| **Jongen et al. (2016)** | $\geq$18 years old  MS confirmed diagnosis (RRMS: 92.86%; SPMS 9.01%)  Taking GA treatment  Relapse free, with stable symptoms  Exclusion: contraindication for GA/sensitivity | 203  GA | Determined by self-report.  Percentage of missed doses in the preceding 14 days at 12 different time points.  Adherent defined as 95% | 52% | - Adherent patients received more home care and informal care than non-adherent patients |
| **Koltuniuk & Rosinczuk (2018)** | $\geq$18 years old  Diagnosis of RRMS only  Taking DMD for at least 6 months | IFN$\beta$-1a (n=43);  IFN$\beta$-1a (n=30);  IFN$\beta$-1b (n=67);  GA (n= 38);  IFN$\beta$-1b (n=29);  Dimethyl fumerate (n=29) | Determined by Self-report  MDR calculated in a 1-month period.  Non-adherent if they missed one or more dose in 28 days or missed at least 25% of the chosen DMT doses | 76.5% | - Most common reasons for non-adherence were forgetting to take the drug, unwilling to take it, it interfered with daily life and dissatisfied with drug |
| **Koskderelioglu et al. (2015)** | $\geq$18 years old  MS confirmed diagnosis (RRMS 83.6%; SPMS 17.4%) | 219  IM IFN$\beta$-1a  IFN$\beta$-1a  IFN$\beta$-1b  GA | Determined by self-report  Criteria for non-adherence was to skip the injections over a 1-month | 53% | - Higher EDSS and depression scores had a significant positive correlation with adherence - Treatment adherence was lower in the group with high education levels |
| **Lahdenpera et al. (2020)** | $\geq$18 years old  Diagnosis of RRMS only,  Taking 1 DMD | 7474  IFNs  GA  Teriflunomide  Dimethyl fumarate | Estimated using the proportion of days covered (PDC).  PDC$\geq$80% was considered adherent | Ranged from 77.2% to 80.3% | - Male sex and older age were associated with better adherence |
| **Li et al. (2020)** | $\geq$18 years old  MS confirmed diagnosis (unspecified type)  Evidence of DMD in 1 year between 2011-2014 | 17,599  All FDA approved DMD’s. | Estimated using the proportion of days covered (PDC).  PDC$\geq$80% was considered adherent | 76.2% at 1 year follow up | - Male gender associated with higher odds of being adherent to MS DMDs - Those with a disability, black ethnicity and on low-income were more likely to be non-adherent than their counterparts - Patients enrolled in enhanced benefit designs were more likely to be adherent than those on standard healthcare plans |
| **Lugaresi et al. (2012)** | $\geq$18 years old  Diagnosis of RRMS only  Eligible for IFN$\beta$-1a | 119  IFN$\beta$-1a | Objective adherence measured through electronic device.  Adherence was calculated as the injections scheduled to be performed in the 12-week time period  $\geq$80% was considered adherent | 88.2% | - No baseline demographic or clinical characteristics predictors of adherence. - Missed injections were accounted for due to forgetfulness (20.7%) and pain at the injection site (3.4%) |
| **Mckay et al. (2017)** | $\geq$18 years old  MS confirmed diagnosis (RRMS: 89.7%; SPMS 9.7%; PPMS: 0.0; CIS: 0.0%; RRMS at onset unknown if progressed: 0.6%)  Taking an injectable DMD during the study period | 485  IM IFN$\beta$-1a  IFN$\beta$-1a  IFN$\beta$-1b  GA | MPR=  Calculated at 3 time-points (baseline, year 1 and year 2)  MPR$\geq$80% was considered adherent | Baseline: 89%  Year 1: 87%  Year 2: 86% | - Non-adherence was associated with a lower EDDS score, disease duration, alcohol dependence, and self-reported cognitive difficulties |
| **Munsell et al. (2016)** | 18-64 years old  MS confirmed diagnosis (unspecified type)  At least one prescription for a self-injectable or oral DMD | 8382  IFN$\beta$-1a  IFN$\beta$-1b  GA  Fingolimod  Teriflunomide  Dimethyl fumarate | MPR calculated.  MPR$\geq$80% was considered adherent | Self-injectable DMD: 54.1%  Oral DMD: 53% | - Adherence was associated with male sex and age groups older than 18-34 years old - Lower likelihood of adherence was associated with depression |
| **Ozura et al. (2013)** | $\geq$18 years old  MS confirmed diagnosis (unspecified type)  Previously treated for at least one month with one first line DMDs. | 299  IFN$\beta$-1a (Avonex) (14.7%)  IFN$\beta$-1a (Rebif) (29.4%)  IFN$\beta$-1b (Betaferon) (32.8%)  IFN$\beta$-1b (Extavia) (1.3%)  GA (21.7%) | Determined by self-report using MSTEQ.  Non-adherent if they missed one or more doses in 28 days or missed at least 25% of the chosen DMT doses | 81.5% adherent with first criterion  (missed one dose)  96.6% adherent for second criterion (missed $\geq$25% injections) | - Most common self-reported reasons for non-adherence were being too busy, not wanting the medication to interfere with other activities, memory problems and side effects |
| **Paolicelli et al. (2016)** | $\geq$18 years old  Diagnosis of RRMS only  Taking IFN$\beta$-1a and  using RebiSmart electronic device for at least 12 months | 384  IFN$\beta$-1a | Objective adherence measured through electronic device  Adherence was calculated as the injections scheduled to be performed in the 12-month time period.  $\geq$80% was considered adherent | 89.3% | - Age (26-40) and EDSS score of <4 was statistically significant between adherent status - Cases of missed doses were related to flu-like syndrome (55.8%), injection-site reactions (20.9%), patient’s choice (16.1%), haematological side effects (7.2%) |
| **de Seze et al. (2012)** | $\geq$18 years old  Diagnosis of RRMS only, an EDSS score $\leq$5.5 and taking DMD for at least 3 months | IM IFN$\beta$-1a (n=93);  IFN$\beta$-1a  (n=32);  IFN$\beta$-1b  (n=41);  GA  (n= 36) | Determined by self-report.  Adherent if did not miss an injection over a 3-month period | 73.8% | - Most common reason for non-adherence was forgetfulness - Adherence was significantly higher in patients who were well informed about disease and treatment |
| **Siegel et al. (2008)** | $\geq$18 years old  MS confirmed diagnosis (unspecified type)  Taking a DMD  Participants could identify a primary caregiver in their life | 54  IFN$\beta$-1a (23.2%)  IFN$\beta$-1a (12.2%  IFN$\beta$-1b (21.4%)  GA (42.9%) | Determined by self-report.  Percentage of adherence was calculated by total doses taken divided by total prescribed dose in the past month.  Adherent$\geq$80% | 85.1% | - Supportive qualities of the caregiver-care recipient relationship prospectively predicted adherent rates |
| **Thach et al. (2018)** | $\geq$18 years old  Diagnosis of RRMS only  Taking an oral or injectable DMD | 489 | Determined by self-report.  Percentage of adherence was calculated by total doses taken divided by total prescribed dose in the past 2 weeks  Adherent$\geq$80% | 92.8% | - Significant predictors of adherence were age, type of DMD (oral or injectable), and DMD experience |
| **Treadaway et al. (2009)** | $\geq$18 years old  Diagnosis of RRMS only  Taking one of the four injectable DMDs for at least 6 months | 798  IFN$\beta$-1a (28%);  IFN$\beta$-1a (19%);  IFN$\beta$-1b (25%);  GA (28%) | Determined by self-report.  Non-adherence defined as missing an injection in the last 4 weeks (collected at three time points; baseline, month 1 and month 2) | Baseline: 61%  Month 1: 63%  Month 2: 64% | - Most common reasons for missing injections were forgetting (58%), did not feel like taking medication (22%) or tired of taking it (16%) - Nonadherent behaviour was also linked to the performance of injection therapy - Perceived effectiveness and benefit of DMD was associated with adherence - Quality of life, depression, hope and satisfaction were associated with adherence status |
| **Tremlett et al. (2008)** | $\geq$18 years old  Diagnosis of RRMS only  Taking DMD for at least 1 month and same drug at follow up (6 months) | 97  IFN$\beta$-1a;  IFN$\beta$-1b;  GA | Determined by self-report  Missed doses in the last 6 months.  ‘Fully adherent’ (missed no doses); ‘missed few doses’ (missed 1–5 doses); ‘missed multiple doses’ (>5 doses) | 6.8% fully adherent  50.5% missed few doses  22.7% missed multiple doses  88% adhered to at least 80% of prescribed doses at each follow up | - Alcohol consumption was positively associated with missed doses - Over one quarter who stopped their DMD had lower educational levels and previous relapses |
| **Turner et al. (2009)** | $\geq$18 years old  MS confirmed diagnosis (unspecified type)  Taking a DMD | 89  IM IFN$\beta$-1a (22.5%);  IFN$\beta$-1a (9.0%);  IFN$\beta$-1b (21.3%);  GA (47.2%) | Determined by self-report  Percentage of  total doses taken divided by total prescribed dose in the past month.  Adherent$\geq$80% | 88.1% at 2-month follow-up  86.3% at 4-month follow-up  87.1% at 6-month follow-up | - Participants who were non-adherent at any follow-up time point endorsed significantly higher injection anxiety at baseline |
| **Zecca et al. (2017)** | $\geq$18 years old  Diagnosis of RRMS only  Taking IFN$\beta$-1a (using RebiSmart for at least 9 month and regularly self-injecting) | 53  IFN$\beta$-1a | Objective adherence calculated as percentage of scheduled injections completed over 9 months and then for 6 months retrospectively using electronic device.  Subjective adherence recorded by patient questionnaire at baseline and 6-month observational period.*  Adherence categories; Low (< 90%), medium (90–99.9%) and high (100%). | Low adherence: 28.3%  Medium adherence: 34.0%  High adherence: 37.7% | - Older age, greater disability, patient’s perception of the importance of ease of use and storage, being informed about RebiSmart, and neurologists’ estimation of adherence were all positively associated with treatment adherence |
